# Effects of phacoemulsification on intraocular pressure and anterior chamber depth

**DOI:** 10.3892/etm.2012.835

**Published:** 2012-11-26

**Authors:** XIN-QUAN LIU, HUA-YING ZHU, JING SU, XIAO-JUN HAO

**Affiliations:** Affiliated Longhua Hospital, Shanghai University of Traditional Chinese Medicine, Shanghai 200032, P.R. China

**Keywords:** cataract, intraocular pressure, glaucoma, lenses, intraocular

## Abstract

The aim of this study was to investigate the effects of phacoemulsification with intraocular lens (IOL) implantation on intraocular pressure (IOP) and anterior chamber depth (ACD) in patients with cataract or cataract associated with primary angle closure (PAC). A total of 361 patients (481 affected eyes) with senile cataract (cataract group) and 44 patients (52 affected eyes) with cataract associated with PAC (cataract with PAC group) underwent phacoemulsification with IOL implantation from July 2005 to May 2007 and were followed up for 3 to 25 months. There was a significant difference between pre-operative and post-operative IOPs (t=9.270, P<0.01) in the cataract group and in the cataract with PAC group (t=3.29, P<0.01). No significant differences were identified in pre-operative IOP (t=−2.437, P>0.05) and the IOP three months after surgery (t=2.154, P>0.05) between the two groups. There was a significant difference between the pre-operative and post-operative ACDs (t=7.781, P<0.01) in the cataract group and in the cataract with PAC group (t=4.528, P<0.01). A significant difference in ACD between the two groups (t=8.325, P<0.01) existed prior to surgery but following surgery, the ACDs of the two groups were not significantly different (t=2.86, P>0.05). Phacoemulsification with IOL implantation has IOP-lowering effects on cataract and cataract with PAC patients. The International Society of Geography and Epidemiology of Ophthalmology classification method for angle closure glaucoma was adopted in our study. Furhter studies are required to prove the safety and mechanism of lowering IOP impact of phacoemulsifation towards PAC glaucoma (PACG).

## Introduction

The incidence of cataract and glaucoma, including primary angle closure (PAC), gradually increases with age. Some patients suffer from both cataract and glaucoma. In the treatment of PAC, phacoemulsification and intraocular lens (IOL) implantation have been used. The occurrence and development of disease risk factors, the anterior chamber angle of PAC and the degree of intraocular pressure (IOP) are reported to be significantly improved by phacoemulsification (1–8).

According to the clinical situation, cataract and glaucoma may be treated by a simple cataract surgery, surgical staging (where cataract surgery is carried out before glaucoma surgery or vice versa) or combined surgery (9). The general risk factors for PAC glaucoma (PACG) include: family history of angle closure (10,11), age (12,13), gender (12–14) and Asian or Inuit descent (15–19). Other risk factors include eye hyperopia (20–22), the perimeter of the shallow anterior chamber (20,22,23), the perimeter of the central shallow anterior chamber (22,24), the steepness of the corneal curvature (25), the thickness of the lens (22,26) and the axial length (22,26,27).

The lens element has been gaining widespread attention. A study has shown that the lens weighs ∼205 mg in 20-year-old individuals and up to 285 mg in 80-year-olds, a growth of >30%. The thickness of the lens at the age of 20 is ∼4 mm and at the age of 65, it may reach 4.8 mm, a 20% increase (28). Therefore, in PAC with cataract, cataract removal by surgery is expected to provide new options on anterior segment structure and its function improvement. We analyzed 481 eyes among 361 patients with cataract and 52 eyes among 44 cataract patients with PAC to observe the effect of phacoemulsification cataract surgery on IOP and central anterior chamber depth (ACD).

## Patients and methods

### General information

From July 2005 to May 2007, 481 eyes in 361 patients (cataract group) undergoing senile cataract phacoemulsification and IOL implantation were investigated. A total of 144 males (193 affected eyes) and 217 females (288 affected eyes) were included.

There were 44 patients (52 eyes) who had cataract with PAC (cataract group), including 18 males (21 eyes) and 26 females (31 eyes) aged 47–94 years old. The mean age was 70.5 years. Eight patients (8 eyes) of cataract patients presented acute attack with an initial IOP>45 mmHg (1 mmHg=0.133 kPa), corneal edema, shallow anterior chamber, dilated and fixed pupil, opaque lens and an anterior chamber angle revealed by gonioscopy to have closed by more than half. These patients were given 20% mannitol, acetazolamide and β-blocker therapy and when the corneal transparency was restored, the IOP returned to normal. Phacoemulsification and IOL implantation was performed after one week.

A total of 36 cases (44 eyes) of cataract associated with chronic angle closure were identified, with an initial IOP higher than normal. They also exhibited a transparent cornea, shallow anterior chamber, normal pupil size, opaque lens and ocular pressure of 21.8–34 mmHg. Gonioscopy revealed that the anterior chamber angle had closed by more than half. Following treatment with acetazolamide and/or β-blocker, the IOP dropped to normal, at which point the patients underwent phacoemulsification and IOL implantation. Follow-up was performed 3–25 months post-surgery, with an average of 13.4 months. This study was conducted in accordance with the Declaration of Helsinki. This study was conducted with approval from the Ethics Committee of the Affiliated Longhua Hospital of Shanghai TCM University. Written informed consent was obtained from all participants.

### Pre-operative examination

In addition to conventional visual function, the IOPs of all patients were measured using a Canon TX-10 tonometer prior to surgery. The results were as follows: 6.50–21.00 mmHg in the cataract group, with an average of 14.86±3.79 mmHg, and 16.20–21.30 mmHg in the cataract with PAC group, with an average of 19.92±3.76 mmHg. The ACD was measured with an Alcon ultrascan A/B ultrasound. The ACD was 1.97–3.25 mm in the cataract group, with an average of 2.38±0.32 mm. The central ACD was 1.29–2.78 mm in the cataract and glaucoma group, with an average of 1.69±0.35 mm. Gonioscopy was used for static checks and pressure checks for both eyes.

### Surgical methods

All surgeries were performed by the same surgeon. Following ocular anesthesia, an incision was made 3.2 mm above the transparent cornea. The anterior chamber was injected with a viscoelastic agent. Following ocular anesthesia, a 3.2mm clear corneal incision was made. The anterior chamber was injected with a viscoelastic agent. Approximately 5 to 5.5 mm diameter continuous curvilinear capsulorhexis was performed. After the lens nucleus was water-separated, phacoemulsification was performed in the capsular bag. The lens cortex was sucked clean using an automatic irrigation/aspiration system. Before and after capsule polishing, the viscoelastic agent was injected into the capsular bag and an artificial lens was implanted into it. The viscoelastic agents were then sucked clean to restore the anterior chamber. All surgeries were successful and no complications occurred.

### Post-operative check

The post-operative check included the slit-lamp examination of anterior segment, keratometry, uncorrected vision acuity and best-corrected vision acuity. The follow-up was conducted at 1 day, 1 week, 1 month and 3 months after surgery to measure the IOP. In the final visit, the anterior chamber angle and depth of both eyes were checked for all cases.

### Statistical analysis

Analysis was carried out using SPSS 11.5 software (SPSS Inc., Chicago, IL, USA). Data were compared between different groups using a t-test. Different time periods were compared using analysis of variance (ANOVA). P<0.01 was considered to indicate a statistically significant difference.

## Results

### IOP

In the cataract group, the mean IOP was 15.24±7.05 mmHg 1 day after surgery; 12.61±4.09 mmHg 1 week after surgery; 12.17±4.22 mmHg 1 month after surgery; 11.83±3.75 mmHg 3 months after surgery and 12.56±3.43 mmHg >3 months after surgery. The IOP pre-surgery was not statistically different from that 1 day after surgery (t=−3.82, P>0.05). The difference between the IOP pre-surgery and 1 week after surgery was statistically significant (F=6.259, P<0.01). The IOP in the cataract with PAC group on post-operative day 1 was 20.61±9.05 mmHg. After 1 week, it was 17.50±5.68 mmHg; after 1 month, 16.80±4.23 mmHg; after 3 months, 17.66±3.56 mmHg and after >3 months, 16.52±3.15 mmHg. The IOP of 8 acute PAC cases on the first post-operative day was more than 40 mmHg, with fibrin-like exudate in anterior chamber, unclear iris texture and corneal edema. After actively reducing the IOP, the inflammation was controlled, the IOP decreased to normal levels and the inflammation subsided. No IOP spike was observed and no IOP lowering therapy was required for the remaining cases in the two groups. The pre-operative and the first day post-operative IOP in each group showed no statiscally significant difference (t=0.514, P>0.05). No significant differences were observed between the two groups in pre-operative IOP (t=−2.437, P>0.05) or IOP from the last visit (t=2.154, P>0.05). However, a significant difference was observed in each group when comparing pre-operative IOP to post-operative IOP 1 week (F=3.290, P<0.01).

### ACD

The ACD axis was found to be 3.04±0.39 mm when a return visit was conducted 3 months after surgery in the cataract group. The difference in the ACD before and after surgery was significant (t=7.781, P<0.01). The anterior chamber shaft depth in the cataract with PAC group ≥3 months after surgery was 2.82±0.41 mm and the difference between the anterior chamber shaft depths before and after surgery was significant (t=4.528, P<0.01). The ACD axes of the two groups prior to surgery were statistically significantly different (t=8.325, P<0.01), whereas the difference between the ACD axes of the two groups following surgery was identified to be of no statistical significance (t=2.86, P>0.05).

## Discussion

Glaucoma of the eye has become the second most prevalent cause of blindness and the primary cause of irreversible blindness (29). Previous studies have confirmed that the damaging effects of PACG occur more rapidly and are more severe than those of primary open-angle glaucoma (POAG) (30,31). In China, PACG is estimated to cause unilateral blindness (visual acuity <3/60 or visual field ≤10°) in 1.5 million individuals and bilateral blindness in another 1.5 million (30).

Elevated IOP caused by the lens element is a primary reason for these types of blindness. With cataract development in the lens, lens thickness and volume increases. The lens also moves forwards, which is knows as a predominant risk factor to pupillary block, during this process. Removal of the lens may effectively prevent the pathogenesis of angle-closure glaucoma (32). The results of the current study reveal that a simple phacoemulsification cataract surgery with PAC may significantly increase the ACD and thus lower IOP. Following successful phacoemulsification surgery, joint capsular bag implantation of the IOL results in a reduced lens thickness and significantly changes the ACD. Several studies have interpreted the mechanism of phacoemulsification and IOP reduction, including the pupillary block relief, iris-lens diaphragm posterior shift, anterior chamber angle widening, and peripheral anterior synechia reopening. Johnstone *et al* proposed a new mechanism of aqueous drainage and IOP. They indicated that the active expansion and retraction of the trabecular meshwork with IOP caused various fluctuations, including blinking and eye movement. The combined Schlemm tube within the valve forms a mechanical aqueous drainage pump system, which provides short- and long-term stability regulations for IOP (33,34). With the collapse and hardening of the Schlemm and trabecular mesh-work tube, the aqueous drainage pumps gradually fail. Thus, the amount of fluid pulsed into the aqueous veins decreases. Furthermore, blood into the Schlemm channel is difficult to observe by gonioscopy. The lens gradually thickens and the relative position of the lens’ front surface moves forward, the trabecular meshwork and Schlemm tube pressure increases and aqueous drainage pump failure increases. Johnstone considered that the failure of the aqueous drainage pump is due to scleral and ciliary muscle attachment. Phacoemulsification and IOL implantation significantly decreases the space occupied by the lens in the anterior segment. Thus, the front surface of the lens moves backwards, the ciliary muscle relaxes and returns to its physiological position, the trabecular meshwork and Schlemm tube stretch and the aqueous drainage pump function is restored. Magnetic resonance imaging has been used to visualize the anterior segment aqueous drainage pumps to validate this theory. Phacoemulsification imaging studies on subjects aged 25, 49 and 74 years have shown that the anatomical location of the aqueous drainage pump changes in all ages, as well as the recovery process following cataract surgery (35,36). Our clinical observations suggest that phacoemulsification is related to cataract IOP control in patients with PAC. The ACD increases and the physiological aqueous drainage pump recovers the anatomical position once aqueous drainage function is restored.

Small incision phacoemulsification of cataracts in cases of acute angle-closure glaucoma and malignant glaucoma, with the lens being part of the pathophysiological mechanism, may effectively reduce IOP, deepen the anterior chamber, open the angle and restore the patient’s visual function. Glaucoma medication may also be reduced. However, a number of patients may forego anti-glaucoma surgery and trabeculectomy surgery to avoid complications, such as excessive filtration surgery, hypotony, shallow anterior chamber, corneal endothelial damage, cystoid macular edema, choroidal leakage, bleeding or scarring of the bleb and poor IOP control, pre-surgery (37).

In the present study, we compared simple cataract surgery with cataract surgery in PAC patients. The IOPs of the two groups and the changes in their central ACD pre-surgery and 1 day after surgery demonstrated no differences. This finding may be related to surgical damage to the blood aqueous barrier, mechanical injury, inflammation of the iris or a surgical residual viscoelastic response of the IOP caused by temporary factors. The condition of acute angle-closure glaucoma patients with grand mal, ciliary body and iris hyperemia, edema and fragile ciliary body did not fully return to normal. Within one week of surgery, a number of patients exhibited inflammatory responses and their conditions became serious. IOP did not decrease and the circumstances of certain individuals had worsened by only 1 day after surgery. However, in time, the inflammatory response subsided; the IOPs of 2 patients 1 week after surgery had decreased significantly compared with their values before surgery and 1 day after surgery. The method currently used as a standard treatment for PAC acute cases is laser peripheral iridotomy (LPI). A randomized study revealed that after 18 months of continuous follow-up, compared with LPI, the effect of cataract phacoemulsification and IOL implantation is more precise in controlling IOP (38). Our observations also confirmed that cataract phacoemulsification and IOL implantation control the effect of cataract IOP associated with acute attack of PAC and IOP pre-operative control to the normal range. Intraocular inflammation requires effective control following surgery to avoid serious reaction. We recommend that for PAC in cataract patients with grand mal, the IOP should be controlled for 1 month after surgery to avoid the emergence of serious reactions. The pre-operative anterior chamber depth was 2.28±0.32 mm in the cataract group and 1.69±0.35 mm in the cataract with PAC group. Post-operative anterior chamber depths were deepened to 3.04±0.39 mm and 2.82±0.41 mm, respectively, which was consistent with the study by Cekic *et al*(39). The ACD axis was >2.5 mm and 98.9% of the angles was greater than 200°, with no possibility of angle-closure glaucoma (40).

Filtering surgery had been recommended for eyes with peripheral anterir synechia of more than 180°. However, for the present study group cases, we considered that cataract plays an important role in the development of angle closure.

Phacoemulsification and IOL implantation remove the triggers of lens expansion, deepen the anterior chamber following surgery, open the angle, normalize the functionality of the damaged trabecular and decrease IOP (40). Patients who use drugs for the effective control of IOP may use this method as a safe and effective surgical choice.

The current study focused on patients with clinically significant cataracts. In PAC or PACG patients, it remains unknown whether the transparent lens should be removed to curb or control IOP PAC progress due to the lack of evidence to support large-scale controlled studies. In the absence of reliable evidence, it appears that PAC for cataract patients is not associated with lens-derived risk factors; thus, its treatment with phacoemulsification cataract extraction surgery should be carefully considered.

## References

[1] Euswas A, Warrasak S (2005). Intraocular pressure control following phacoemulsification in patients with chronic angle closure glaucoma. J Med Assoc Thai.

[2] Hayashi K, Hayashi H, Nakao F, Hayashi F (2001). Effect of cataract surgery on the intraocular pressure control in glaucoma patients. J Cataract Refr Surg.

[3] Lai JS, Tham CC, Chan JC (2006). The clinical outcomes of cataract extraction by phacoemulsification in eyes with primary angle-closure glaucoma (PACG) and coexisting cataract: a prospective case history. J Glaucoma.

[4] Tham CC, Kwong YY, Leung DY (2008). Phacoemulsification versus combined phacotrabeculectomy in medically controlled chronic angle closure glaucoma with cataract. Ophthalmology.

[5] Suzuki R, Kuroki S, Fujiwara N (1997). Ten year follow up of intraocular pressure after phacoemulsification and aspiration with intraocular lens implantation performed by the same surgeon. Ophthalmology.

[6] Tong JT, Miller KM (1998). Intraocular pressure change after sutureless phacoemulsification with foldable posterior chamber lens implantation. J Cataract Refr Surg.

[7] Tennen DG, Masket S (1996). Short and long term effect of clear corneal incisions on intraocular pressure. J Cataract Refr Surg.

[8] Issa SA, Pacheco J, Mahmood U, Nolan J, Beatty S (2005). A novel index for predicting intraocular pressure reduction following cataract surgery. Brit J Ophthalmol.

[9] Johnson SM (2009). Cataract Surgery in the Glaucoma Patient.

[10] Leighton DA (1976). Survey of the first-degree relatives of glaucoma patients. Trans Ophthalmol Soc UK.

[11] Perkins ES (1974). Family studies in glaucoma. Br J Ophthalmol.

[12] Seah SK, Foster PJ, Chew PT (1997). Incidence of acute primary angle-closure glaucoma in Singapore. An island-wide survey. Arch Ophthalmol.

[13] Bengtsson B (1981). The prevalence of glaucoma. Br J Ophthalmol.

[14] Wolfs RC, Grobbee DE, Hofman A, de Jong PT (1997). Risk of acute angle-closure glaucoma after diagnostic mydriasis in nonselected subjects: the Rotterdam Study. Invest Ophth Vis Sci.

[15] Bourne RR, Sorensen KE, Klauber A, Foster PJ, Johnson GJ, Alsbirk PH (2001). Glaucoma in East Greenlandic Inuit - a population survey in Ittoqqortoormiit (Scoresbysund). Acta Ophthalmol Scan.

[16] Foster PJ, Baasanhu J, Alsbirk PH, Munkhbayar D, Uranchimeg D, Johnson GJ (1996). Glaucoma in Mongolia. A population-based survey in Hovsgol province, northern Mongolia. Arch Ophthalmol.

[17] Congdon N, Wang F, Tielsch JM (1992). Issues in the epidemiology and population-based screening of primary angle-closure glaucoma. Surv Ophthalmol.

[18] Nguyen N, Mora JS, Gaffney MM (1996). A high prevalence of occludable angles in a Vietnamese population. Ophthalmology.

[19] Lai JS, Liu DT, Tham CC, Li RT, Lam DS (2001). Epidemiology of acute primary angle-closure glaucoma in the Hong Kong Chinese population: prospective study. Hong Kong Med J.

[20] Van Herick W, Shaffer RN, Schwartz A (1969). Estimation of width of angle of anterior chamber. Incidence and significance of the narrow angle. Am J Ophthalmol.

[21] Lowe RF (1970). Aetiology of the anatomical basis for primary angle-closure glaucoma. Biometrical comparisons between normal eyes and eyes with primary angle-closure glaucoma. Br J Ophthalmol.

[22] Lavanya R, Foster PJ, Sakata LM (2008). Screening for narrow angles in the Singapore population: evaluation of new noncontact screening methods. Ophthalmology.

[23] Friedman DS, Gazzard G, Foster P (2003). Ultrasonographic biomicroscopy, Scheimpflug photography and novel provocative tests in contralateral eyes of Chinese patients initially seen with acute angle closure. Arch Ophthalmol.

[24] Alsbirk PH (1975). Anterior chamber depth and primary angle-closure glaucoma. I An epidemiologic study in Greenland Eskimos. Acta Ophthalmol (Copenh).

[25] Sihota R, Lakshmaiah NC, Agarwal HC, Pandey RM, Titiyal JS (2000). Ocular parameters in the subgroups of angle closure glaucoma. Clin Exp Ophthalmol.

[26] George R, Paul PG, Baskaran M (2003). Ocular biometry in occludable angles and angle closure glaucoma: a population based survey. Br J Ophthalmol.

[27] Lavanya R, Wong TY, Friedman DS (2008). Determinants of angle closure in older Singaporeans. Arch Ophthalmol.

[28] Levin LA, Nilsson SFE, Hoeve JV, Wu S, Kaufman PL, Alm A (2011). Adler’s Physiology of the Eye.

[29] Resnikoff S, Pascolini D, Etya’ale D (2004). Global data on visual impairment in the year 2002. Bull World Health Organ.

[30] Foster PJ, Johnson GJ (2001). Glaucoma in China: how big is the problem?. Br J Ophthalmol.

[31] Bourne RRA, Sukudom P, Foster PJ (2003). Prevalence of glaucoma in Thailand: a population based survey in Rom Klao District, Bangkok. Br J Ophthalmol.

[32] Ge J, Guo Y, Liu YZ (2001). Clinical evaluation of phacoemulsification on management of angle closure glaucoma. Chinese Journal of Ophthalmology.

[33] Johnstone MA (2004). The aqueous outflow system as a mechanical pump: evidence from examination of tissue and aqueous movement in human and non-human primates. J Glaucoma.

[34] Johnstone MA (2006). A new model describes an aqueous outflow pump and explores causes of pump failure in glaucoma. Glaucoma.

[35] Strenk SA, Strenk LM, Guo S (2006). Magnetic resonance imaging of aging, accommodating, phakic and pseudophakic ciliary muscle diameters. J Cataract Refract Surg.

[36] Strenk SA, Strenk LM (2007). In vivo MRI. Visualizing the haptics. Eye World.

[37] Zang J, Bao JL, He LR (2003). Clinical study on phacoemulsification with IOL implantation on acute angle closure glaucoma. Chinese Journal of Ophthalmology.

[38] Lam DS, Leung DY, Tham CC (2008). Randomized trial of early phacoemulsification versus periphera iridotomy to prevent intraocular pressure rise after acute primary angle closure. Ophthalmology.

[39] Cekic O, Batman C, Totan Y (1998). Changes in anterior chamber depth and intraocular pressure after phacoemulsification and posterior chamber intraocular lens implantation. Ophthalmic Surg Lasers.

[40] Aung T, Nolan WP, Machin D (2005). Anterior chamber depth and the risk of primary angle closure in 2 East Asian populations. Arch Ophthalmol.

